# Patient Experience of Living With Hemophilia A: A Conceptual Model of Humanistic and Symptomatic Experience in Adolescents, Adults, and Children

**DOI:** 10.36469/001c.123374

**Published:** 2024-10-18

**Authors:** Zalmai Hakimi, Rakhee Ghelani, Linda Bystrická, Nana Kragh, Patrick Marquis, Jameel Nazir, Nadine McGale

**Affiliations:** 1 Sobi, Stockholm, Sweden; 2 Modus Outcomes, Cambridge, Massachusetts, USA

**Keywords:** hemophilia A, signs and symptoms, health-related quality of life, qualitative research, patient outcome assessment, treatment experience, humanistic burden

## Abstract

**Background:** People living with hemophilia A face challenges impacting their daily lives despite treatment innovations. Previous studies have explored perceptions and treatment experiences; however, there is a lack of an evidence-based, comprehensive model to identify concepts (clinical, physical, and psychological functioning) relevant for people with hemophilia A (PwHA). **Objectives:** The aim of this qualitative study was to address the question: What is the humanistic and symptomatic experience of adolescents, adults, and children living with hemophilia A and what is the impact of hemophilia A on their quality of life? **Methods:** Participants, identified through patient associations in the UK, were male PwHA and caregivers of male PwHA receiving prophylactic treatment. Qualitative research was conducted involving semistructured telephone interviews with PwHA and caregivers between April 2020 and September 2020 in the UK. Standard analytical techniques of conceptual model development were used. **Results:** Of 30 participants, 23 were PwHA and 7 were caregivers. A conceptual model was produced describing patient experience of symptoms, physical functioning, treatment experiences, and the impact of symptoms and treatment on daily lives. Participants reported hemophilia-related symptoms, including bleeding, pain, and joint stiffness, as well as difficulties engaging with social and leisure activities. They also reported protection from bleeds provided by their treatment, relief from symptoms, and the resultant sense of normality. Concepts were broadly relevant across all age groups; however, psychological impacts were reported only by adult PwHA, and caregivers reported impacts related to outdoor activities, play, and education. Participants indicated that their ideal treatment would be delivered orally. **Discussion:** This study highlights the range of symptoms experienced by PwHA across a broad range of age groups, thus enabling the evaluation of relevant concepts across different stages of life. The research supports development of a conceptual model documenting symptoms, impacts, and treatment experience relevant to PwHA. **Conclusion:** Insights gathered through the interviews and resulting conceptual model support development of new therapies to address the physical and social challenges identified by PwHA and highlight a need for novel hemophilia A treatments that can ease treatment administration, provide adequate level of protection, and enable life to be lived normally.

## BACKGROUND

Hemophilia A is a rare congenital coagulation disorder caused by a deficiency in factor VIII (FVIII), with an estimated prevalence at birth of 24.6 cases per 100 000 males for all severities.^1,2^ The treatment landscape and principles have evolved over the years, and an increasing emphasis is placed on understanding the experiences of people with hemophilia and their families to reduce their treatment and disease-associated burdens.^1^

Various therapies, such as standard half-life, extended half-life (EHL), and non-factor therapies, are currently used as prophylaxis treatment across Europe. Although associated with a higher treatment burden compared with on-demand treatment, prophylaxis throughout childhood is suggested to result in the preservation of musculoskeletal function.^3^ Prophylaxis with EHL products has reduced the number of infusions required to achieve protection from bleeding as well as providing effective management of pain arising from bleeding.^1,4^ This approach has also demonstrated meaningful improvements in health-related quality of life (HRQoL),^5^ with a better health state (a higher mean index score in the HRQoL questionnaire, EuroQol 5-dimensions [EQ-5D]) reported in adults treated on prophylaxis, when compared with those treated on-demand and with short-term prophylaxis.^6^

However, people living with hemophilia continue to face many challenges impacting their daily lives despite the innovations in care. People with hemophilia in Nordic countries have a higher likelihood of use, higher volume of use, and longer duration of pain medications than matched population controls.^7^ In addition, people living with hemophilia in the United Kingdom had the lowest mean EuroQol 5-dimensions 5-level instrument (EQ-5D-5L) index score across the EU5 (France, Germany, Italy, Spain, United Kingdom).^8^ A significant reduction in HRQoL scores measured by EuroQol visual analog scale, EQ-5D-5L, and Patient Reported Outcomes, Burdens, and Experiences (PROBE) scores was also seen in males with mild or moderate hemophilia compared with people with no bleeding disorders in an international study.^9^

During studies involving interviews with people with hemophilia, challenges related to mobility have been highlighted as a consequence of bleeds, such as issues in the joints or upper limb following a bleed,^10,11^ as well as people with hemophilia reporting limitations in joint function affecting the ability to walk and climb stairs.^12^ Other reported hemophilia-related burdens include a negative impact on social activities, holidays, caring for children, and daily activities; an impact on work and education; and restricted career options.^10–13^

Previous studies reporting perceptions of people with hemophilia on the potential drivers for treatment choice have also noted the importance of administration frequency, factor VIII storage during travel, treatment side effects, and mode of administration.^14,15^ Furthermore, concerns regarding aging, declining eyesight, and other health issues that would pose challenges for self-injection have been raised by older people with hemophilia and other inherited bleeding disorders.^16^

The psychological impact of hemophilia A has also been studied, highlighting reports of people with hemophilia concealing their diagnosis from others, feeling overwhelmed, and worrying about treatment and the future in general.^13,17^ Children and their caregivers have indicated a need to adapt sports choices and noticed a feeling of being socially isolated/distanced from peers.^10^ In addition, some people with hemophilia worry about passing their illness on to their sons.^13,17^

Previous studies have explored perceptions and treatment experiences of people with hemophilia A (PwHA) and B. However as the treatment landscape evolves,^18^ there is a need to update the perspective of people with hemophilia on both the disease and treatment burden, and address the lack of a comprehensive, evidence-based model to conceptualize the patient humanistic and symptomatic experience of hemophilia A. This study aimed to understand the humanistic and symptomatic experience and burden of living with hemophilia A from the perspective of adolescents/ young adults, older adults, and caregivers of children with hemophilia A, along with the impact on HRQoL and unmet treatment needs. By developing a conceptual model offering insights into the patient-specific experience, this study adds to the current evidence.

## METHODS

### Study Design

This was a noninterventional, descriptive, cross-sectional qualitative research study involving semistructured interviews with PwHA and caregivers was conducted between April 2020 and September 2020 in the United Kingdom.

### Participants and Ethical Considerations

Convenience sampling was utilized to identify participants. Participants were identified through patient associations in the United Kingdom, who advertised the study among its members; the recruitment process was coordinated by a healthcare research company, with interested participants screened for eligibility. Male PwHA were included if they were aged 12 years or older, self-reported a diagnosis of hemophilia A, and were receiving prophylactic treatment. Also included were the caregivers of male PwHA aged under 12 years with a diagnosis of moderate to severe hemophilia A currently receiving prophylactic treatment.

Interested participants who fulfilled the above criteria were asked to provide written informed consent. The Health Research Authority ethics tool, used to determine whether National Health Service (NHS) Research Ethics Committee (REC) review was required for the study, was completed and indicated that NHS REC approval was not necessary given the scope of this non-invasive research and non-NHS recruitment route.

### Interview Conduct

Telephone interviews lasting approximately 60 minutes were conducted by experienced qualitative researchers. Interviews explored the participant experience of living with hemophilia, focusing specifically on symptoms and their impacts on daily life, and treatment, using a semistructured interview guide that contained 3 concept elicitation sections related to the patient experience of (1) symptoms, including experiences of global and joint pain, (2) impacts of living with hemophilia, and (3) treatment-related experiences. Structured questions within the interview guide related to treatment experience included the following: “What do you consider are the biggest benefits of your hemophilia treatment?”, “What are the biggest drawbacks or downsides of your hemophilia treatment?”, and “What would your ideal treatment look like?” Insights regarding the experiences of children were gathered through interviews conducted with their caregivers. Interviews were recorded, anonymized, and transcribed verbatim.

### Data Analysis

Transcripts were coded in ATLAS.ti software using an open, inductive, thematic approach with coding conducted in parallel for each of the groups of interest (adolescents/young adults, older adults, and caregivers of children). The first transcript was independently coded by 2 researchers; any coding inconsistencies were discussed and reconciled. Researchers met regularly to discuss and adjust coding guidelines when necessary.

Standard analytical techniques of conceptual model development were used.^19–21^ Codes and, where necessary, quotations were compared with the rest of the data and in an iterative process inductively categorized into higher-order overarching categories reflecting their conceptual content such as codes, subdomain level 2, subdomain level 1, domain, and overarching domain.

Saturation was assessed by ordering interviews chronologically, then grouping interviews into 6 groups of 5 interviews. Concepts emerging in each group were compared sequentially to assess whether saturation had been reached. Saturation was indicated when no new concepts emerged. Considering the detailed and granular nature of coding in this study, saturation analysis was conducted on a sub-domain level.

## RESULTS

### Demographics and Health Status of PwHA

A total of 30 participants were recruited comprising 17 adolescent/young adults (12-35 years), 6 older adults (>35 years) and 7 caregivers of children (<12 years) with hemophilia A. The majority of PwHA were White/Caucasian. Moderate/ severe hemophilia was reported by 90% of people, and the remaining 10%, who reported mild hemophilia, reported moderate/severe hemophilia at eligibility screening during recruitment. Most PwHA had their last bleed within 1 year, and the number of bleeds per year on current treatment varied within and between age groups. The mean age of adolescents/young adults, older adults, and children was 23.0, 45.7, and 8.4 years, respectively (**Table 1**).

**Table 1.** Demographics and Health Status of Male People With Severe Hemophilia A^a^

	**Adolescents/Young Adults, 12-35 y (n = 17)**	**Older Adults, >35 y (n = 6)**	**Children, <12 y (n = 7)^b^**
Mean age (range), y	23 (13-34)	45.7 (37-55)	8.4 (3-12)
Gender, n (%)			
Male	17 (100.0)	6 (100.0)	7 (100.0)
Ethnicity, n (%)			
White/Caucasian	16 (94.1)	6 (100.0)	7 (100.0)
Other	1 (5.9)	0	0
Self-reported severity of hemophilia, n (%)			
Mild	2 (11.8)	0	1 (14.3)
Moderate	1 (5.9)	1 (16.7)	0
Severe	14 (82.3)	5 (83.3)	6 (85.7)
Level of clotting factor, n (%)^b^			
0	3 (17.6)	0	2 (28.6)
≤1	9 (52.9)	6 (100.0)	4 (57.1)
3-8	3 (17.6)	0	1 (14.3)
Missing data	2 (11.8)	0	0
Treated at a hospital, n (%)	7 (41.2)	3 (50.0)	4 (57.1)
Presence of inhibitors, n (%)	2 (11.8)	0	0
Use of factor/concentrate before activity/procedure, n (%)	11 (64.7)	5 (83.3)	5 (71.4)
Use of factor/concentrate on regular schedule, n (%)	14 (82.4)	6 (100.0)	5 (71.4)
Diagnosis, n (%)			
Birth to ≤1 y	14 (82.4)	4 (66.7)	7 (100.0)
>1-3 y	3 (17.6)	2 (33.3)	0
Last bleed, n (%)			
Within 1 year	13 (76.4)	6 (100.0)	5 (71.4)
Within 2 y	2 (11.8)	0	1 (14.3)
Within 5+ y	2 (11.8)	0	1 (14.3)
Bleeds per year with current treatment, n (%)			
0-1	6 (35.3)	0	2 (28.6)
2-3	4 (23.5)	1 (16.7)	0
4-5	2 (11.8)	1 (16.7)	3 (42.9)
6-10	1 (5.9)	4 (66.7)	1 (14.3)
>10-20	3 (17.6)	0	1 (14.3)
Missing data	1 (5.9)	0	0
Frequency of treatment medication per week, n (%)			
On-demand	1 (5.9)	0	0
1-2 per week	4 (23.5)	2 (33.3)	1 (14.3)
3-4 per week	9 (52.9)	4 (66.7)	4 (57.1)
1 per day	1 (5.9)	0	0
1 per week	0	0	1 (14.3)
1 every 2 weeks	1 (5.9)	0	1 (14.3)
Missing data	1 (5.9)	0	0

^a^90% of people reported moderate/severe hemophilia and the remaining 10% who reported mild hemophilia reported moderate/severe at eligibility screening during recruitment.

^b^As reported by caregivers.

### Conceptual Model

**Figure 1.**
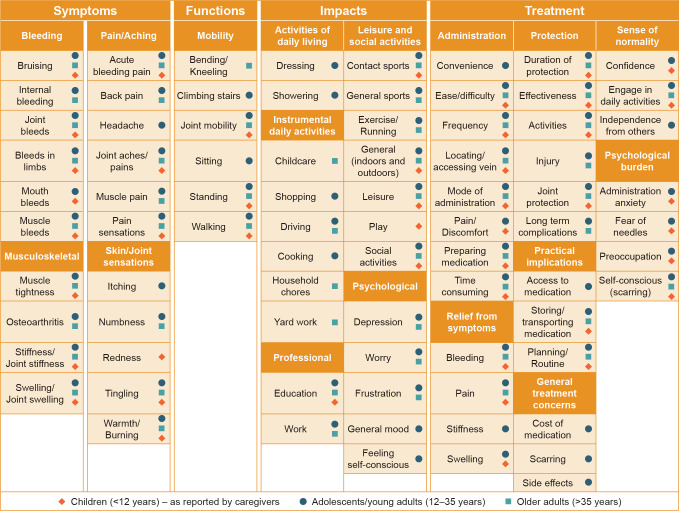
Conceptual Model of Patient Experience and Breakdown of Concepts Reported for Each Age Group Note: Concepts could correspond to both positive and negative experiences.

Concept elicitation analysis identified 361 unique codes from the 30 interview transcripts, which were inductively categorized into higher-order overarching categories, resulting in 4 overarching domains that described different aspects of participants’ experience of hemophilia including symptoms, functions, impacts, and treatment. The breakdown of the model, showing overarching domain, domain, and subdomain level 1, based on the age groups of interest (adolescents/young adults, older adults, and children) is presented in **Figure 1**.

**Symptoms**: The overarching symptoms were categorized into 4 domains related to hemophilia disease manifestation–bleeding, musculoskeletal, pain/aching, and skin/joint sensations (**Figure 1**).

When discussing bleeding episodes, participants described bleeds largely in terms of location (eg, internal bleeding, bleeds in limbs, mouth bleeds, joints bleeds, muscle bleeds) and the propensity to bruise.

Participants across all 3 age groups reported musculoskeletal symptoms, including joint swelling, muscle tightness, and stiffness in joints. The adolescents/young adults and older adults age groups reported osteoarthritis as a result of damage to their joints following recurrent bleeds; this was not observed in children.

Pain was reported by all participants, especially pain related to acute bleeding episodes and joint damage (joint aches/ pains). One patient was quoted:

I have had this my whole life and I can certainly remember, like, having quite a lot of pain with bleeds and things and obviously I’m pretty sure they would always just give me treatment and painkillers and things.

A small number of PwHA in the adolescents/young adults age group reported experiencing headaches, although this was not apparent in the other age groups. Sensations in the skin and joints that participants associated with bleeds were described as tingling and a hot or burning sensation (**Figure 1**).

**Functions:** The second overarching domain of functions contains the domain of mobility. Participants reported impairment of function in terms of reduced mobility, which was affected during an acute bleeding episode and was also chronically persistent. All 3 groups reported issues with the range of mobility in their joints (including ankles and elbows), limitations relating to their ability to stand for long periods of time and impaired walking ability. A patient was quoted:

It’s internal bleeding, predominantly to my joints. Which, you know, left untreated, becomes very, very painful, results in a lot of swelling and reduced movement.

Additionally, adolescents/young adults experienced difficulties sitting for long periods of time and climbing stairs, whereas older adults reported difficulties bending and kneeling (**Figure 1**).

**Impacts:** The overarching domain of impacts contains 5 domains. Participants spoke about a range of hemophilia-related impacts to their daily lives, including those which were detrimental to them psychologically and professionally, along with others that impaired their ability to participate in activities.

Participants reported impacts on daily activities necessary for fundamental functioning, including difficulty with self-care tasks such as dressing and showering (adolescent/young adults).

Difficulties engaging with leisure and social activities were reported by all age groups. A patient was quoted:

It makes it harder to do stuff than the average person, for example, playing sports, I have to pick less contact sports in order to ensure my safety. For example, I can’t play football because then I hurt my ankles and when I get hurt it’s really bad and it takes a long time to recover.

Caregivers of children specifically referenced their child’s limitations relating to playing with their peers. The social impact of living with hemophilia was described by one patient who was quoted:

Some people get the wrong idea of what it can do. So, it has destroyed, sort of, relationships, friendships and such, you know, where people can’t fathom why I can’t do certain things or take part in certain activities, etc.

Within the leisure and social activities domain, the general (indoors and outdoors) concept was used when participants were unable to specify what activities they felt were restricted.

Participants in the adolescents/young adult and older adult age groups reported restrictions with various essential daily activities that would allow them to live independently, including childcare, shopping, driving, cooking, and home maintenance. A quote from another patient stated: “…impact on the illness is concerned, is a big impact as far as walking, doing daily chores around the house, daily… just basically my standard of living has taken a massive hit due to internal bleeding caused by hemophilia.”

The psychological impact domain included concepts associated with feelings of depression, worry, frustration, general mood, and hemophilia-related self-consciousness. The general mood concept was used when PwHA described mood swings or changes in mood as a direct result of hemophilia. A caregiver noted that a patient “…would be having to lay on the sofa and do nothing all day which is not what he likes doing. It can lead to frustration and anger that you’ve got this condition as well.”

Participants across the 3 age groups described disruptions in their education due to hemophilia. Adolescents/young adults and older adults reported that hemophilia limited their work opportunities and the types of careers they could aspire to (**Figure 1**).

**Treatment:** The final overarching domain of treatment contains 7 domains. When considering their treatment, participants reported positive concepts relating to the treatment-related protection from bleeds, relief from symptoms (including bleeding, swelling, and pain), and the resultant sense of normality. Participants also indicated concerns associated with treatment administration as well as practical general concerns, and a psychological burden (**Figure 1**).

Treatment administration concepts corresponded to both positive and negative experiences, with some participants describing the frequency of treatment administration as beneficial and others perceiving it as a drawback. Moreover, adolescent/young adult participants described difficulties accessing medication, along with general treatment-related concerns around scarring, side effects, and the costs incurred by the UK NHS for providing long-term treatment.

Adolescent/young adult PwHA and the caregivers of children described a treatment-related psychological burden, which could be driven by needle phobia or the persistent mental load (preoccupation). Participants from all age groups reported feeling self-conscious due to the scarring caused by treatment administration.

The practical implications of a lifelong treatment dependency experienced by all participants included the need to plan treatment administration (planning/routine), and complications with storing and transporting medication, especially while traveling.

Participants spoke positively about the level of protection from bleeds and joint damage offered by their treatment. This facilitated engagement with daily activities and allowed participation in activities including sports. The duration and efficacy of protection offered by treatment was a concept agreed by all age groups.

Treatment enabled participants to live life with a sense of normality, and both adolescents/young adults and children described gaining confidence from the treatment. A patient was quoted: “I’m not dependent on other people to care for me, which is one of the things that I get from my medication, is that independence to be able to treat myself” (**Figure 1**).

As part of the interview, participants were asked, “What would your ideal treatment look like?” The majority reported their preferred mode of administration would be an oral medication or a pill (**Table 2**). Other concepts the participants felt were important were related to the frequency and time taken for treatment administration, and the level of protection (and its duration) provided by the treatment.

**Table 2.** Ideal Treatment Identified by Participants (N = 30)^a^

**Ideal Treatment**	**n (%)**
Mode of administration: pill/oral	13 (43)
Gene therapy	7 (23)
Less frequent administration	7 (23)
Longer lasting protection	5 (17)
Mode of administration: similar to insulin pen	5 (17)
Quick administration	4 (13)
Cure	3 (10)
Symptom improvement: instant relief	3 (10)
Protection for sports	3 (10)
Lead normal life	2 (7)
Mode of administration: not injection	2 (7)
Symptom improvement: joint damage	2 (7)
Easy disposal	1 (3)
Effective	1 (3)
Mode of administration: implants	1 (3)
Symptom improvement: pain	1 (3)
No side effects	1 (3)
Pre-prepared injection	1 (3)
Symptom improvement: stiffness	1 (3)

^a^Multiple responses were allowed per person.

### Data Saturation

The first 3 groups of interviews (n = 15) produced 78/85 (92%) of the concepts, and, correspondingly, all 17 of the domains presented in the model. New concepts emerging after the first 15 interviews added granularity to the comprehensive conceptual model but did not introduce any new domains within any of the 4 overarching domains. The final 2 groups (n = 10) of interviews added only 3 new concepts to the model: muscle tightness, play, and leisure. Muscle tightness added granularity to the “musculoskeletal” domain. Leisure and play expanded the “leisure and social activities” domain and were only reported by caregivers of young children with hemophilia. The saturation analysis supported the comprehensiveness and granularity of the conceptual model.

## DISCUSSION

This study provides an evidence-based conceptualization of the patient humanistic and symptomatic experience of living with hemophilia A, reflecting experiences across all stages of life. People with hemophilia and caregivers of children with hemophilia described the experience of key symptoms associated with hemophilia, the functional mobility impact of those symptoms and the wider impacts patients experience in their daily lives. This study adds in-depth insights into the patient experience of prophylactic treatment of hemophilia A, describing considerations from the perspective of PwHA and caregivers of children with hemophilia A including administration and the protection offered by treatment.

Treatment-related concepts were identified as relevant across all different age groups; prophylaxis treatment is recommended for people with severe phenotype hemophilia, with children ideally starting treatment prior to 3 years of age.^1^ Consistent with previous findings, PwHA and caregivers considered symptom relief and practical considerations, such as storing and planning for treatment, as important.^6^ PwHA and caregivers have reported challenges associated with coordinating treatment schedules and medication/supply management.^22^ Adolescents/young adults and caregivers noted a psychological burden associated with treatment with fear of needles and persistent mental load. These considerations could impact the choice of treatment, including the administration routes available, and whether additional psychological support is needed for PwHA within these age groups.

PwHA provided insight into their ideal treatment, with concepts considered most important including preferred pill or oral mode of administration and others related to the treatment administration (quick and less frequent), and the level of protection provided (longer lasting). Similar preference for treatment with less invasive administration has been noted by PwHA and caregivers in previous studies,^15,23^ as well as desire for a reduction in administration frequency.^15^ These insights highlight unmet needs for PwHA regarding current treatment.

Caregivers reported hemophilia affecting their children’s education due to missing days off school for treatment and missing out on outdoor activities and play with peers. For male children and adolescents with congenital bleeding disorders, school attendance and participation in sports are positively associated with HRQoL.^24^ The main differences across the 3 age groups were linked to the concepts related to the activities and psychological impact domains. Caregivers did not report concepts related to activities of daily living or broad daily activities as it is likely their children were too young to be engaged in performing these independently.

The psychological impact of living with hemophilia was described in terms of feelings of depression and worry, frustration and feeling self-conscious, which are consistent with other interviews where people with hemophilia, including those with other bleeding disorders, reported feeling misunderstood, rejected, or mistreated.^13,16^ Previously, people with hemophilia have also highlighted other psychological aspects such as avoiding revealing their disease for fear of being isolated and repelled by society, and feeling that hemophilia affects their personal identity with participants proposing constraints in physical activities as a primary reason for their identity challenges.^25,26^ An observational study found that higher anxiety and depressive symptoms were more likely to be associated with more urgent hospital visits due to hemophilia, more bleeding episodes, more affected joints and pain, as well as worse levels of perceived functionality and HRQoL in the previous year.^27^ Our study findings highlight the need to address the psychological impact of hemophilia has on PwHA and that, with development of newer treatment, current goals of people with hemophilia are health equity and a “hemophilia-free mind.”^28,29^ The ability to perform normal activities without fear of pain or bleeding, and thus improved quality of life, is a quality in treatment preferred by both PwHA and caregivers.^23^ In the current study, psychological impacts were reported only by adult PwHA (adolescents/young adults as well as older adults) and not reported by caregivers of children with hemophilia A, which is likely due to the significantly smaller sample size for caregivers compared with the sample for younger adults. Therefore, concepts reported here could be expanded with studies using a larger sample size.

The study included PwHA providing an in-depth insight into experiences of prophylactic treatment. Inclusion of a broad range of age groups supported the evaluation of relevant concepts across different age groups and life stages. Previous patient-reported outcome studies have been conducted before the marketing authorization of EHL or non-factor therapies with a larger proportion of people treated on-demand. The strength of our study is that PwHA were evaluated in the current treatment landscape.

However, the study was conducted in the United Kingdom with a limited sample size, which may have limited the ability to draw generalizable conclusions; therefore, future research could aim to include a larger population of all ages of interest. In addition, further research should be conducted to confirm the cross-cultural relevance of the study by including a more diverse population as experiences of hemophilia and of treatment, including accessibility and availability, may differ between countries. As the qualitative research was conducted with participants from patient associations, there is the possibility of selection bias, and caution should be taken when interpreting results in a wider context, due to the possibility that these participants may have been better informed than the average PwHA. The timing of the interviews coinciding with the early stages of COVID-19 restrictions should also be taken into consideration, as this may have influenced participants’ experiences of hemophilia and, therefore, their responses during the interviews. Indeed, during the COVID-19 pandemic, people with hemophilia receiving prophylactic treatment expressed concern over treatment supply and were reported to be anxious and frightened.^30^ We did not aim to elicit all possible challenges experienced by PwHA but to use a semistructured interview approach to explore concepts that are likely to be relevant for PwHA and caregivers.

The data presented in this study are qualitative, as such future work could involve collating quantitative data via use of a survey in order to inform the generalizability of the current data and further explore the experiences of people living with hemophilia A.

## CONCLUSION

The study highlights the range of symptoms experienced by adolescents/young adults, older adults, and children living with hemophilia A. The research supported the development of a comprehensive conceptual model documenting the symptoms, impacts, and treatment experience relevant to PwHA. Treatment-related concepts were identified as relevant across all different age groups, with the main differences in concepts related to the activities and psychological impact domains. Important concepts for ideal treatment from a PwHA/caregiver perspective included administration mode and frequency, and the duration of protection offered by treatment. As new treatments for hemophilia A become available, insights from PwHA and caregivers, gathered through the interviews conducted during this study and the resulting conceptual model, can inform patient-centered outcomes research and support advancements in treatments to address the physical and social challenges identified by PwHA and address unmet needs. Findings from this qualitative study highlight a need for novel treatments for hemophilia A that can ease treatment administration, provide adequate level of protection, and enable life to be lived as normal.

### Ethics Approval and Consent to Participate

The Health Research Authority ethics tool was completed which indicated that given the scope of this research and recruitment route, no NHS Research Ethics Committee (REC) approval was necessary. Signed informed consent was obtained from PwHA or guardians of PwHA prior to participation.

### Data Availability

Data access will be granted in response to qualified research requests. All requests are evaluated by a cross-functional panel of experts within Sobi, and a decision on sharing will be based on the scientific merit and feasibility of the research proposal, maintenance of personal integrity, and commitment to publication of the results. To request access to study data, a data sharing request form (available on (http://www.sobi.com/) should be sent to medical.info@sobi.com. Further information on Sobi’s data-sharing policy and process for requesting access can be found at: https://www.sobi.com/en/policies.

### Competing Interests

Nadine McGale and Patrick Marquis are employees of Modus Outcomes, a consultancy company that received funding from Sobi for this research. Rakhee Ghelani was also an employee of Modus Outcomes at the time that the trial was conducted. Linda Bystrická, Nana Kragh, Jameel Nazir and Zalmai Hakimi are employees of Sobi.

### Author Contributions

All authors were involved in the conception and design of the study. N.M.G., R.G. and P.M. were involved in analyzing the data associated with the study. All authors were involved with drafting and revising the manuscript and provided the final approval of the manuscript. All authors agree to be accountable for all aspects of the work.
